# The Chinese Experience of Rapid Modernization: Sociocultural Changes, Psychological Consequences?

**DOI:** 10.3389/fpsyg.2016.00477

**Published:** 2016-04-05

**Authors:** Jiahong Sun, Andrew G. Ryder

**Affiliations:** ^1^Culture, Health, and Personality Lab, Centre for Clinical Research in Health and Department of Psychology, Concordia UniversityMontreal, QC, Canada; ^2^Culture and Mental Health Research Unit, Jewish General HospitalMontreal, QC, Canada

**Keywords:** Chinese modernization, rapid sociocultural change, individualism, depression, urban and rural

## Abstract

Mainland China has undergone profound changes dating back to the nineteenth century, including a contemporary period of rapid modernization that began in the 1980s. The result has been dramatic social, cultural, and economic shifts impacting the daily lives of Chinese people. In this paper, we explore the psychological implications of sociocultural transformation in China, emphasizing two central themes. First, rising individualism: findings from social and developmental psychology suggest that China’s rapid development has been accompanied by ever-increasing adherence to individualistic values. Second, rising rates of depression: findings from psychiatric epidemiology point to increasing prevalence of depression over this same time period, particularly in rural settings. We argue that links between sociocultural and psychological shifts in China can be usefully studied through a cultural psychology lens, emphasizing the mutual constitution of culture, mind, and brain. In particular, we note that the link between social change, individualism, and rising mental illness deserves careful attention. Our review suggests that shifting values and socialization practices shape emotion norms of concealment and display, with implications for depressive symptom presentation. The challenge comes with interpretation. Increasing prevalence rates of depression may indeed be a general response to the rapidity of sociocultural change, or a specific consequence of rising individualism—but may also result from increasingly ‘Western’ patterns of symptom presentation, or improvements in diagnostic practice. We conclude by considering the challenges posed to standard universal models of psychological phenomena.

## Introduction

“At no time and in no circumstances should a Communist place his personal interests first; he should subordinate them to the interests of the nation and of the masses. Hence, selfishness, slacking, corruption, seeking the limelight, and so on, are most contemptible, while selflessness, working with all one’s energy, whole-hearted devotion to public duty, and quiet hard work will command respect”.

(Mao, 1938/1966, p. 269)

“In a factory with one thousand or ten thousand people, to have the boss discover you is very hard. You must discover yourself. You must develop yourself. To jump out of the factory, you must study… If you are waiting for your company to lift you up, you will grow old waiting”.

(A 17-year-old female factory worker; Chang, 2008, p. 174)

Although both quotations touch on the self, the differences are stark. In the former, the self is subordinated to the greater interest of the collective—seeking the advancement of personal interest is selfish and corrupting. In the latter, the self is empowered and motivated to achieve a better future, and indeed is enjoined to do so. Between Chairman Mao’s call for collectivism in the 1930s and the insistence of individualism in the early 21st century, China underwent tremendous social, economical, and political change. In terms of the sheer number of people affected, these upheavals may well be unprecedented in human history. There is every indication that the blistering pace of change continues to this day, deeply affecting the everyday lives of over one billion people.

In this paper, we aim to explore the psychological consequences of rapid sociocultural change in China. We begin with a brief review of these changes since the 1980s, establishing both its scale and its speed compared to other countries over the same time period. We argue that rapid sociocultural change has implications for cultural psychology—given the mutual constitution of culture and mind (Markus and Kitayama, 1991; Shweder, 1991), profound cultural change ought to have deep psychological consequences. We then briefly consider the definitions of modernization, followed by a review of studies converging on the theme of rising individuality in China. Next, we discuss the psychological implications of rising individuality using examples of changes in emotion norms and symptom presentation. Lastly, zeroing in on the mental health consequences of rapid sociocultural change, we examine evidence for the increasing prevalence of mental illness. We argue that the adverse effects of rapid changes are widespread, with a disproportionate impact on rural populations. At the same time, we note that rapid sociocultural change has also brought changes in how symptoms of mental illness are presented. We conclude by reflecting on the implications of sociocultural change for cultural and cultural-clinical psychology, followed by a brief discussion of potential future directions for researching the psychological consequences of sociocultural change.

## China’s Rapid Sociocultural Change, From 1980 to 2010

A 30-year period represents but a tiny fraction of China’s 5,000-year civilization. Yet, rapid sociocultural change is an essential part of how people in China understand their own recent history, stretching back for at least two centuries. From the opening of the treaty ports (1842) and the anti-foreign Boxer Rebellion (1900), to more recent social movements such as the Great Leap Forward (1958–1960) and the Great Proletarian Cultural Revolution (1966–1976), the Chinese people are no strangers to social change, or to sudden reversals of fortune. The year 1978 marked yet another new beginning: Deng Xiaoping re-opened China’s doors to the influence of the wider world, engendering a series of economic, social, and political changes, and transforming large swaths of the country from poverty to prosperity.

In a sociological review of contemporary China’s ‘social transformation’, Xie (2011, pp. 14–15) states that, “China’s ongoing social transformation since the late twentieth century is no less consequential for the long-term course of world history than events commonly considered as historical watersheds, such as the Renaissance that began in fourteenth-century Italy, the Protestant Reformation in sixteenth-century Germany, or the Industrial Revolution in eighteenth-century Britain.” In support of this bold claim, he provides three evidence-based examples. First, he notes that per-capita gross domestic product (GDP), net of inflation, increased at a rate of 6.7% per year between 1978 and 2008—contrasted with the ‘golden age’ of American industrialization, 1.5% per year between 1860 and 1930. Second, he reports a considerable increase in education level, particularly at the postsecondary level, with 0.8% of the population aged 25 to 29 holding a postsecondary degree in 1982, compared with 12% in 2005. Finally, he argues that China had essentially completed the demographic transition typical of advanced societies by 1990–1995, from high fertility and high-mortality to low fertility and low mortality, during this time. He adds that this shift is important, as it tends to promote investment in human capital, which in turn contributes to economic growth.

These economic and demographic shifts are interwoven with numerous sociocultural changes, including the shift from a majority rural to majority urban population, the long-term effects of the One Child Policy^1^ on family organization, and rapid increases in consumption of both home-grown and international electronic media^2^. While other countries may have undergone similarly rapid economic and sociocultural transformations in their histories, dating back at least to the original Industrial Revolution in the UK, the sheer size of China’s population ensures an unprecedented scale. We begin by considering the implications of sociocultural change for cultural psychologists given the mutual constitution of culture and mind. Then, we explore some specific shifts in psychological functioning that have accompanied these sociocultural changes. Finally, we consider the implications of these shifts for mental health and well-being.

## Why Should Cultural Psychologists Care About Social Change?

The core claim of cultural psychology is not simply that ‘culture matters’, but more importantly that culture and mind “make each other up” (Shweder, 1991). Culture and mind mutually constitute and influence one another, and neither is viewed as the ultimate source of the other. Here, we emphasize a definition of culture that incorporates both beliefs and practices. Indeed, Markus and Hamedani (2007) argue for a *sociocultural* approach, encouraging the field to view culture as both conceptual and material. Culture thus includes meanings, ideas, and attitudes that live ‘in the head’, and also cultural products, institutional practices and systems, and interpersonal interactions that exist ‘in the world’. We believe that China’s rapid change is best understood from a mutual constitution perspective. Not only do these rapid changes influence individual people’s thoughts, behaviors, and so on, widespread changes in the latter in turn shape the meanings and practices of the larger Chinese society.

In the past few years, the idea of mutual constitution has been extended to incorporate brain as another level (Kitayama and Uskul, 2011; Ryder et al., 2011). Culture, mind, and brain are hence understood as a single dynamic system with multiple levels; causation cannot be reduced to any one level, and changes at one level affect the others (Ryder and Chentsova-Dutton, 2012). For example, rapid change in the economic structure of a society accompanied by shifts away from old value systems (culture-level) may increase stress for some people, leading to heightened worry about the future (mind-level) and increasing cortisol levels (brain-level). Increased cortisol levels over a prolonged period can in turn have behavioral consequences, and a society may shift further in response to widespread worries about the future. Of course, these complex effects can also happen in a positive direction. The important point is that researchers should anticipate changes at the brain- and mind-levels as part of China’s rapid sociocultural transformation.

Culture is not static. Even under normal conditions, cultural meanings and practices are never mere replicas of the past: they shift and change over time, from generation to generation, shaped by social, political, and economic forces (Gjerde, 2004). Given that culture changes, and given the mutual constitution of culture and mind, we therefore propose that the rapid sociocultural transformations in Chinese society are accompanied by psychological changes, specifically including a new emphasis on individualistic values. We also raise the possibility that changes in economic status and cultural values may have contributed to greater mental health concerns, as documented by psychiatric surveys and evidence of unevenly distributed mental health problems in rural China. We choose to focus on these two domains as they are among the most widely studied phenonema that can be plausibly linked to social change in China.

Individualism-collectivism by cultural and cross-cultural psychologists, with a formidable body of research associating collectivism with East Asian societies and individualism with the modernized West. Meanwhile, the mystery of low depression rates in China, the potential explanation of these rates as consequences of cultural variation in symptom presentation, and observed changes in this pattern over time, comprise one of the most well-known set of findings in cultural psychiatry (Ryder and Chentsova-Dutton, 2012). It is important, moreover, to consider potential mental health consequences when exploring the psychological implications of potentially disruptive processes. We conclude by briefly arguing that engagement with sociocultural change may force behavioral scientists to reckon with the historical instability of supposedly universal constructs, but at the same time opens exciting avenues for understanding psychological phenomena in cultural-historical context.

## Modernization and the Rise of Individualism in China

Social scientists have long regarded value change as central to modernization, and have paid considerable attention to the association between societal modernization and the adoption of individualistic values (e.g., Inkeles and Smith, 1974; Hofstede, 2001; Greenfield, 2009). We begin by considering briefly the definition of modernization and its relation to westernization. Next, we review existing ‘Western’^3^ theories and findings linking modernization and individualistic values. We then turn to recent empirical evidence demonstrating the rising importance of individualism in China. In closing, we consider the implications of rapid modernization and value change on mental health functioning, before pointing to future considerations when studying the impact of sociocultural change on psychological processes.

### Modernization and Westernization

The common view of modernization refers to the process of becoming modern, involving the transformation of a traditional or less economically developed society to a modern, industrialized society (Armer and Katsillis, 2002). China’s rapid sociocultural change involves exactly this sort of shift. For some theorists, however, the term ‘modernization’ also carries the expectation that, since modernization was first experienced and completed in the West, a similar trajectory will be followed in other parts of the world (Scott and Marshall, 2009). In other words, China is not simply modernizing—it is, by definition, Westernizing. This second perspective has been criticized for ethnocentrism and failing to consider the wider role of globalization (Bruce and Yearley, 2006). Moreover, it also stands at odds with the experiences of industrialized non-Western societies, such as Japan, Korea, Israel, and Turkey.

For instance, in a cross-temporal analysis of individualism and collectivism, Hamamura (2012) found that both Japan and the US experienced a similar rise of individualism since the 1950s, marked by urbanization, shrinking family size, and an increasing divorce rate. Nonetheless, people in Japan were more likely to retain traditional collectivistic values compared to those in the US: increasing individualism has not necessarily meant decreasing collectivism, at least not in a zero-sum way. The retention of collectivism in Japan has consequences. For example, Americans, but not Japanese, have reported a decline in trusting others over time. In addition, the importance of social obligation, social harmony, and social contribution for Japanese, but not Americans, has actually increased over time, suggesting that collectivistic values continue to be important in modern Japanese society. Japan has not simply followed the process of modernization in a formulaic way, thereby becoming a Western country. Instead, modernization-induced value change can co-exist with important aspects of traditional culture.

In addition, modernization in contemporary developing countries, profoundly influenced by globalization, is fundamentally different from how the West was industrialized, which to a large extent relied on capital formation and colonial expansion (Wen, 2007). Yan (2010) argues that the shift toward greater individualism in Western Europe began under conditions of relative aﬄuence and political democracy, where individual rights were protected and satisfaction of material needs was not the primary goal of societal development. In contrast, China’s shift started with widespread poverty and economic shortage, situated in a very different political system with very different views on individual freedoms, and with accumulation of material wealth as the primary goal. From different points of departure we observe different trajectories of modernization.

Our view is that modernization and westernization are neither completely independent processes, nor are they synonymous. Instead, we expect that modernization, in most parts of the world, unfolds in a context of global and Western influences as much as it is shaped by traditional cultural values and practices. To this end, we consider the rise of individualism in China as shaped by the combined forces of rapid modernization and westernization.

### Models of Modernization and Individualism

Several theories across the social sciences have addressed the impact of rapid sociocultural change on values and personality characteristics (e.g., Kahl, 1968; Guthrie, 1977; Yang, 1981). From sociology, Inkeles and Smith (1974) proposed the concept of individual modernity, arguing that a particular set of psychological characteristics, including attitudes, values, and ways of feeling and thinking, prepare a person to be an effective member of a modern society. In six developing countries, they identified a coherent set of characteristics, including: (a) being receptive and open to new experiences, innovation, and change; (b) being proactive in acquiring information and facts; (c) thinking about the present or future instead of dwelling on the past; and (d) being confident in one’s ability to achieve one’s goals. From this perspective, a modern person is a highly autonomous, open-minded, motivated, and flexible—and, importantly, is an informed participant in society with a clear sense of personal efficacy (Inkeles, 1983).

From cross-cultural psychology, Hofstede’s (2001) comparative study of work-related values in more than 40 national cultural contexts provides another source of empirical evidence linking modernization and individualism at the societal level. Hofstede (1984) described four dimensions of cultural values: power distance; uncertainty avoidance; masculinity; and individualism. In seeking to explain systematic cultural variations in these value dimensions, Hofstede found a striking correlation of 0.84 between individualism and GDP per capita, an economic measure frequently used to index societal modernization. His finding implies that we should expect individualistic values to emerge in tandem with economic development.

Distinctions between societal- and individual-level values deserve further explication as they reflect different sets of phenomena. Culture- or society-level values concern norms that are emphasized and pursued to varying degrees in different societies (Schwartz, 2011). They reflect the nature of societies or groups and ways of attaining fundamental societal goals. In contrast, individual-level values are aspects of personality that reflect a range of motivationally distinct goals for basic human living (Schwartz, 2006). In our view, individual-level and societal-level values are intricately connected and mutually influenced by sociocultural changes. That Chinese *society* is rapidly modernizing might have implications for societal-level individualism, which in turn may stimulate further changes in personal individualism. Personal value changes taking place in a given sociocultural context may be understood at least in part as the consequence of societal modernization.

Greenfield’s (2009) theory of social change and human development marks an important advance in thinking about the impact of societal-level change on individual-level development. Her approach begins with the consideration of two contrasting prototypical social organizations: Gemeinschaft (small-scale rural community) and Gesellschaft (large-scale urban society). Centering on the idea of social adaptation, Greenfield posits that collectivism and individualism, or interdependence and independence, partially describe appropriate sociocultural adaptions to rural versus urban living environments. For example, she argues that sharing among the extended family, a key feature of collectivism, helps one to adapt to the daily practices of a small-scale rural community. In contrast, the need for personal privacy, an important aspect of individualistic values, helps one to adapt to large-scale urban ecology, such as living in houses with separate bedrooms. Greenfield further posits that as one type of society moves toward the other prototype, it is likely that learning will take place and people will gradually shift their values in order to maximally adapt to the new environment. The theory of social change and human development predicts a greater emphasis on individualistic values as people move from rural to urban settings and as societies move toward greater modernization. We should therefore expect the increasing centrality of individualism to accompany China’s rapid modernization.

### Evidence of Rising Individuality in China

Shortly after the advent of China’s open-door policy, Chinese scholars and educators noted changes in young people’s attitudes or awakened sense of individualism, including self-awareness, independence, and a growing concern for personal well-being (Yu, 1997; Bai, 1998). Sun and Wang (2010) assessed value differences in four generations of Shanghai residents. Compared to older generations, people in the younger generations were more likely to nominate self-development as being the most important life priority, whereas political engagement was least important. In addition, more than half of the participants between the ages of 14 and 18 strongly agreed with the statement that one should live according to one’s own style regardless of what others think. Note that the centrality of family remained an important value across age groups. Findings from this study demonstrated a shift toward individualistic values, although traditional values were not completely abandoned.

The World Values Survey (WVS)^4^ conveys a similar message. The WVS is a global research project developed primarily by sociologists to study changing values and their impact on society. The two recent waves of the WVS in China (2007 and 2012) incorporated a 10-item measure of Schwartz’s 10 basic human values, including those reflecting collectivistic or individualistic tendencies (Schwartz, 2006). Compared to 2007, Chinese respondents in 2012 were less likely to endorse collectivistic values, such as traditionalism and conformity; also, they were less likely to reject individualistic values, such as hedonism and power. Although these values data are only available for the most recent two WVSs, shifts in the importance of individualistic and collectivistic values in just 5 years demonstrate a striking effect of rapid sociocultural change at the individual level.

In addition to values changes, Steele and Lynch (2013) found that individualism has an increasingly strong association with subjective well-being (SWB) in China. In their analyses, demographic indicators commonly correlated with individualistic values, such as personal income, employment status, and self-rated health, demonstrated a strengthening association with SWB over time. In contrast, measures reflecting collectivistic sentiment, such as national pride and support for collectivistic policies (e.g., equal income for all), became less important in predicting personal well-being. Although measures of individualistic and collectivistic orientations both predicted SWB to varying degrees, the former has gradually become more central in fostering a sense of well-being. Why has this occurred?

One possibility is that culturally salient values are transmitted from one generation to the next (Kroeber and Kluckhohn, 1952) through children’s socialization. Recent reports from developmental psychology studies point to important shifts in Chinese parenting. For example, themes of raising happy, healthy, and autonomous children emerged from narratives of 24 Chinese mothers of middle school students (Way et al., 2013). Another recent study showed that mothers in Beijing, compared to immigrant Chinese mothers in the US, adopted a more Western pattern of affection-based interaction with their toddlers (Wang, 2013). Indeed, the meaning of shyness may itself be changing. In traditional Chinese cultural contexts, shy-sensitive children are perceived as well behaved; here, shyness signifies social accomplishment and maturity rather than social withdrawal or disinterest (King and Bond, 1985). Where shy-sensitivity is an accepted and at least somewhat valued character trait, shy-sensitive children are more likely to receive social support, which in turn helps them integrate better socially and form meaningful relationships (Chen, 2000). Notable shifts are observed, however, when one compares school-aged cohorts from 1990, 1998, and 2002. The positive association between shy-sensitivity and adjustment reported in 1990 was no longer statistically significant for same-aged children in 1998 (Chen et al., 2005). For the 2002 cohort, moreover, shy-sensitivity was positively associated with self-reported depression and peer rejection, and negatively associated with teacher-rated competence. Shy-sensitivity, once an acceptable and even positive personality trait in many Chinese contexts, has become associated with social disadvantage and disapproval.

A second possibility is that China’s rapidly emerging economy is accompanied by a parallel emergence of individualism. Indeed, Kraus et al. (2012) proposed a social cognitive theory of social class to account for differences in the ways people from lower- and upper-class contexts think about the self, perceive the world, and relate to others. The authors argued that social cognitive patterns in people of lower socioeconomic status (SES) involve a more contextual and relational style, in contrast to the individualistic orientation characteristic of those of higher SES. Indeed, Grossmann and Varnum (2015) found that changing levels of SES was the most robust predictor of shifting patterns of individualism in the US over the last 150 years. Research on this topic, largely conducted with American samples, has generally supported the link between social class and social cognitive style. For example, people from lower SES background were more likely to show engagement behavior in a brief encounter with a stranger, such as head nods and laughs, whereas those from higher SES background were more likely to display disengagement behavior, such as self-grooming and doodling (Kraus et al., 2009). In another study, compared to Americans and people with higher SES, Russian participants and those of lower SES were more holistic when deciding the extent to which individual characters/attitudes versus the surrounding environment/social norms influenced the protagonist’s action (Grossmann and Varnum, 2011).

These findings show that SES is strongly connected to values and how one relates to others, suggesting that rising SES is an important factor in understanding the growing importance of individualism in China. For example, one study examined different levels of narcissism in young adults from urban and rural settings (Cai et al., 2012). Narcissism refers to a self-aggrandizing, entitled, dominant, and manipulative orientation (Campbell et al., 2006), and arguably represents the extreme end of individualism. In this study, one-child status, higher SES, and urban living were significantly associated with higher levels of narcissism in young adults. This finding suggests that the rising importance of individualism may be unevenly distributed among sociodemographic groups.

In addition, if values are shaped by sociocultural contexts and if Chinese urban and rural societies have experienced varying degrees of modernization, then we should expect variations in these values in residents from urban and rural areas. Compared to rural parents, urban parents reported greater changes in work-related opportunities, self-improvement, and high-technology experience, and their children received lower levels of parental control and a greater encouragement of independence, compared to children from rural settings (Chen et al., 2010). A third group, urbanized families, was included in a follow-up study (Chen and Li, 2012). Urbanized families are former rural residents who live on what used to be the outskirts of urban centers. Due to China’s rapid urban expansion and development, these former rural residents were re-categorized as urban residents. Compared to rural families, urbanized families showed a pattern of parenting similar to urban parents, such as encouraging initiative taking in children. Moreover, children from urbanized families received higher peer-rated sociability-assertiveness scores than their rural peers. These findings are consistent with the perspectives of Greenfield (2009) and Kraus et al. (2012), discussed earlier, and point to the powerful influence of social context on parenting attitudes in China and its subsequent effect on the socialization of children’s autonomy and independence.

Taken together, findings from the recent literature demonstrate that aspects of traditional parenting practices emphasizing compliance, self-control, and cooperation are giving way to assertiveness, autonomy, and initiative taking. Moreover, shifts in parental attitudes and goals may be more prominent in urban than rural settings, suggesting that SES might be an important driver of increasing individualism. Thus, future studies should not only directly test the relation between SES and individualism in China, but also seek to explore the underlying mechanisms through which economic status influences individualistic values and practices.

### Traditional Values in Modern China

China’s rapid sociocultural transformation, marked by modernization and economic growth, accompanies parallel changes in aspects of traditional values and practices—particularly those pertaining to education and parenting. Drawing on recent findings from China, as well as Japan and Mexico, we argue that traditional values do not simply disappear with the rise of individualistic values; rather, they co-exist and mutually reinforce one another.

Traditional practices can persist in various ways, even when the circumstances giving rise to these patterns no longer hold. For example, Talhelm et al. (2014) tested the hypothesis that people from predominantly rice-farming provinces in China, compared to those from wheat-farming provinces, continue to show a higher degree of interdependence as a legacy of the cooperation and coordination needed for effective rice farming. Data consistent with this prediction were obtained from samples of students who did not themselves have farming experience. Moreover, the effect remained after controlling for regional GDP per capita, and even persisted when comparing rice versus wheat regions of the same province. Not only does a particular history of traditional subsistence practices continue to influence the self-construals of people living in modern China, this work raises questions about whether we should expect ‘traditionalism’ or ‘modernization’ to look the same in different regions of this vast country.

Research from Japan, another East Asian society that modernized relatively quickly, further supports the idea that traditional values can persist. In his cross-temporal analysis, Hamamura (2012) demonstrated that traditional cultural meanings and practices play an important role in shaping the effects of modernization. For instance, although mass education is an expected outcome of modernization, the ways with which education is delivered can vary. Japanese educators were more likely to approach learning by emphasizing hard work and self-improvement, whereas Western traditions of education place a greater emphasis on hypothesis-testing and self-directed learning. Shimizu et al. (2014) found that the majority of Japanese mothers in 2008 and 2009 slept with their babies in the same room, a traditional practice that was just as prevalent as it had been several decades previously. Interestingly, mothers who reported co-sleeping frequently expressed discrepancies between their values of maternal/infant independence and the co-sleeping arrangement, reflecting persistent social expectations on women to perpetuate certain traditional practices. More importantly, these findings show that modernized societies do not necessarily involve a straightforward, uncontested relation between modern versus traditional meanings and practices (see also Manago and Greenfield, 2011).

This is not to downplay the extent to which values have changed over time, nor to say that all traditional values show equal persistence. Xu and Hamamura (2014) found that, compared to 50 years ago, Chinese participants reported that materialism, individualism, and human rights have become increasingly important. Some traditional cultural values, such as family relations, friendship, and patriotism, have maintained their perceived importance whereas other values, such as traditional ways of living and Confucian ethics, have declined. Interestingly, the same researchers found that Google Ngram analyses, a method of studying popularity of topics by looking at usage of word frequency in published materials over a period of time, yielded a divergent pattern of rising interest in traditional topics (e.g., Confucian ethics).

Zeng and Greenfield (2015) reported similar findings, also using Ngram. Individualistic values, indicated by words such as ‘choose’, ‘compete’, and ‘autonomy’, showed an upward trend over the last 40 years, and were positively associated with indices of social change. In contrast, some collectivistic values, indicated by words such as ‘communal’, ‘obedience’, and ‘sacrifice’, declined in frequency. However, words reflecting other aspects of collectivistic values, such as ‘obliged’ and ‘give’, continued to be important. These findings not only point to the multifaceted composition of individualism and collectivism, they also suggest persistence of traditional values in a context of rapid modernization. Indeed, the authors argued for the adaptive functioning of co-existing individualistic and collectivistic values, adding that modernization has taken place at different rates in different parts of China.

We therefore caution against viewing individualism and collectivism as necessarily incompatible. Instead, our review suggests that modernization is a non-uniform process that impacts different strata of Chinese society at varying speed, and that increasing individualism does not necessarily signify the end of traditional meanings and practices. We argue that future cultural psychology studies would benefit from a more nuanced and balanced approach by asking the following questions: how do individualism and collectivism manifest themselves in a rapidly modernizing society; how do traditional values shape and influence modernization, and vice versa; and finally how does an individual person separate, integrate, or negotiate potential disagreement involving different sets of meaningful practices?

## The Chinese Experience of Rapid Modernization: A ‘Double-Edged Sword’

What are the consequences of rising individualism and rapid social change more generally for the psychological well-being of the Chinese people? If there are costs, are these due to individualism *per se* or to the rapidity of the shift away from traditional values? The answers to these questions must be considered cautiously, as links between modernization, individualism, and psychological well-being are not straightforward. Although China continues to have one of the lowest prevalence rates for depression worldwide (Bromet et al., 2011), these rates appear to have rapidly increased. As the increase in depression prevalence has occurred over the same time period as the increase in individualism, it is tempting to identify the former as a consequence of the latter. We must consider, nonetheless, a number of other possibilities including: changes in diagnostic category and research methodologies; changes in symptom presentation that facilitate diagnosis; and changes in willingness to report certain symptoms. All of these possibilities might themselves be consequences of rapid sociocultural change, as all could involve a shift toward Westernized norms. It is thus exceedingly difficult to determine whether observed changes are due to rapid and/or widespread change in general, a move toward a value system with detrimental consequences for mental health, or adoption of improved assessment methods that more effectively capture the suffering that was always present.

Rather than solving this conundrum—an impossible task in any case, given the evidence available—we instead reflect on how these different possibilities appear to have interacted over the past several decades in China. We begin with research cataloging changes in well-being and depression over the past several decades, before turning to the demographic inequities regarding who has suffered the most from sociocultural transformation. With these general findings in mind, we conclude by considering the potential influence of shifting emotion norms on the experience and expression of depression.

### Modernization, Well-Being, and Depression

When measured at the level of the person, values associated with individualism tend to be positively associated with well-being and healthy psychological adjustment. For instance, there is evidence from China supporting self-determination theory, which posits that endorsement of intrinsic life goals (e.g., personal growth, community building, and satisfying relationships) promotes psychological well-being (Ryan and Deci, 2000). For example, in a study conducted with Chinese and North American children, autonomy supportive parenting in both groups was associated with greater endorsement of intrinsic life goals in children, who in turn reported better mental health (Lekes et al., 2010).

On the other hand, studies of subjective well-being have revealed declining life satisfaction in China from 1990 to 2010, despite enjoying an average economic growth of ≥8% per year (Brockmann et al., 2008; Wang and VanderWeele, 2011; Easterlin et al., 2012). Several changes may be responsible for this apparent paradox. First, the transition from a centrally controlled economy to a market economy has given rise to striking socioeconomic inequities, providing greater opportunities for unfavorable comparisons regardless of absolute improvements in circumstances (Brockmann et al., 2008). Secondly, we argue that a growing emphasis individualism also means that one is ultimately responsible for his/her own well-being, which may in turn lead to more dysfunctional self-focused ruminative thinking during times of failure. Placing a much greater emphasis on individualism may leave the modern person more vulnerable when responding to unfavorable situations.

Studies from psychiatric epidemiology generally support the increased prevalence of depression over the last decades. Two early psychiatric surveys reported extremely low rates of depression in China, where 0.045 and 0.083% of those surveyed in 1982 and 1993 had a lifetime affective disorder (Twelve-Region Psychiatric Epidemiological Study Work Group, 1986; Zhang et al., 1998). In the early 2000s, mental health surveys sponsored by the World Health Organization (WHO) reported a 3.5% lifetime prevalence of major depressive disorder in metropolitan China—a striking increase in little over a decade (Shen et al., 2006; Lee et al., 2007). A recent meta-analysis of epidemiological studies of depression published from 2001 to 2012 showed converging prevalence rates with the WHO surveys, suggesting that at least 3.3% of the Chinese population will experience depression at some point in their lifetime (Gu et al., 2013).

### Social Inequality and Its Implications for Mental Health

When considering how modernization affects different parts of China, we argue that the effects of rapid sociocultural change are unevenly distributed, with China’s rural population being more negatively affected compared to their urban peers. To understand China’s urban and rural differences, Chinese citizens are categorized from birth as non-agricultural (urban) or agricultural (rural) residents under the household registration system called *hukou*. The *hukou* system, established in 1950, serves as the backbone of China’s institutional structure and allows the government to better control internal migration (Wang, 2004). Generally speaking, people cannot acquire permanent legal residential status, and its associated benefits, outside the area covered by their *hukou*. Given that China’s internal labor migration almost always originates from rural areas, the vast majority of rural migrant workers reside in urban centers as temporary residents. In other words, with rare exceptions, people do not change their *hukou*. Many scholars (e.g., Chan, 2009; Whyte, 2010) consider the *hukou* system to be a major source of inequality in China, used to justify differential rights, benefits, and privileges given to its citizens. Indeed, China’s urban and rural divide pertains to multiple levels of disparity, including economic, political, sociocultural, and health/mental health.

For example, recent psychiatric surveys consistently reported higher rates of psychological distress in rural China across the lifespan. For example, compared to urban students, students from rural settings reported higher levels of depressive symptoms (Luo et al., 2008). Similarly, psychiatric epidemiological surveys reported that adults and elderly adults in rural areas have a higher prevalence of psychiatric disorders compared to urban residents (Li et al., 2008; Ma et al., 2010). Further studies have reported elevated psychological distress in rural-to-urban migrant workers, and the children and parents of those workers (Silverstein et al., 2006; Ye and Pan, 2011; Zhong et al., 2013; Chen et al., 2014; Ding and Bao, 2014).

As part of the sociocultural transformation of the last several decades, China has experienced the largest internal migration in human history. Much of this movement has been from the countryside to the city, and has largely been driven by the search for a better economic future (Chan, 2013; Ding and Bao, 2014). The apparent benefits, however, appear to come at a psychological cost. As of 2010, the National Bureau of Statistics of China (2010) estimates that 230 million Chinese citizens—approximately one in six people—are migrant workers. These migrant workers are commonly referred to as the ‘floating population’ due to their classification as temporary urban residents under China’s *hukou* system. Due to social-structural, cultural, and often educational barriers, rural-to-urban migrant workers are unable to fully integrate into urban society and are rarely given the same benefits urban residents receive, such as employment and education opportunities (Myerson et al., 2010).

The literature generally underscores the negative consequences of migrant worker status within China. Some studies have reported increased psychological distress in migrant workers, and such findings are often discussed in the context of migration stress, marginalization, and discrimination (Wong et al., 2008; He and Wong, 2013). Recently, Zhong et al. (2013) conducted a meta-analysis of studies using the Symptom Checklist-90-R (SCL-90-R) to assess psychological outcomes in migrant workers. The meta-analysis revealed that, compared to Chinese norms on the SCL-90-R, migrant workers experienced worse mental health on almost all symptom dimensions. Yet these effects are at least somewhat mitigated for people who perceive their migration as bringing meaningful financial support to their families, or as an opportunity for personal development (Wong and He, 2008). This finding suggests that future studies should attend to the migration goals of rural-to-urban workers, and the extent to which these goals are being met or are perceived as likely to be met in the future.

As most migrant workers cannot move their families to the cities, their children, parents, and sometimes spouses remain in the countryside, and are often referred to as the ‘left behind’ population. According to survey results from 2010, a total of 61 million children were left behind in rural China, 50 million of whom were under the age of 14 (The All-China Women’s Federation, 2013). One apparent consequence of parental migration is disturbed psychological well-being in these children. Compared to rural children living with both parents, those with one or both parents absent were three times more likely to experience depression (He et al., 2012). Moreover, parental absence is associated with a tendency to be reluctant when communicating with these children (Ye and Pan, 2011). Loneliness was the most common word these children used to describe their feelings, increasing risk for depression through social isolation. In sum, an impoverished familial environment, irregular diet, poor hygiene, increased domestic labor, decreased personal safety, and loosened parent–child contact, put the ‘left-behind’ children at risk for psychological maladjustment (Ye and Pan, 2011; Ding and Bao, 2014).

Another group suffering from loneliness is the elderly population in rural China (Chen et al., 2014). Compared to their urban peers, older people in rural China are at an elevated risk for depression (Su et al., 2012; Zhang et al., 2012). The disruption of traditional support from family members, such as living in a multigenerational household, confers potential psychological problems for the elderly. With China’s mass internal migration, an increasing number of older people now live alone or with their ‘left-behind’ grandchildren without the presence of their adult children (Silverstein et al., 2006). Elderly people living alone reported higher levels of depression symptoms and a lower of life-satisfaction compared with those living in multigenerational households.

### The ‘Unleashing’ of Emotion in China

A central claim of cultural psychology is that emotions, integral to the self-concept, are shaped by cultural context. In other words, there are cultural differences in the prevalence, patterns, responses, potentials, and determinants of emotions (Mesquita and Walker, 2003). Furthermore, contemporary theorizing on culture and emotion holds that emotions are emergent properties of larger sociocultural as well as immediate contextual factors. If the experience and expression of emotion is to a certain extent embedded in a given sociocultural context, what kind of impact does sociocultural change and its associated values shifts have on emotion norms? We believe that changes in cultural values, specifically the rising importance of individualistic values and shifts in socialization norms, shape emotions in the direction of more expressivity. Moreover, changing emotion norms of concealment and display entail further implications for symptom presentation in emotional disorders, such as those characterized by depression or anxiety (Chentsova-Dutton et al., 2014).

In the late 1970s and 1980s, the low rate of depression in Chinese populations was one of the first systematically reported cultural variations in prevalence rates. This does not signify a lack of suffering. Kleinman (1982) noted that while fewer than 1% of psychiatric outpatients were diagnosed with depression, 31% were assigned a diagnosis of ‘neurasthenia’ (i.e., *shenjing shuairuo*, translated as “weakness of nerves”). The category of neurasthenia includes many experiences similar to those associated with depression, but with a marked emphasis on somatic symptoms, such as headache, fatigue, muscle pain, sleep disturbance, and so on (Lin, 1989). Kleinman’s (1982) landmark study of 100 Chinese patients diagnosed with neurasthenia found that most of these patients met standard Western diagnostic criteria for depression, but at the same time presented a different clinical picture, minimizing depressed mood while emphasizing physical symptoms. He argued that at this time, after years of political unrest, emotional disclosure was considered inappropriate and potentially dangerous. In this view, physical symptoms and a neurasthenia diagnosis represented somatic ‘idioms of distress’, ways of communicating distress in a socially and politically acceptable manner (Kleinman and Kleinman, 1995).

Kleinman’s study was closely followed by the open-door policy of Deng Xiaoping, triggering the latest wave of rapid sociocultural change, with a pronounced impact on the material and psychological life of people in China (Lee, 2011). One consequence is that people in the younger generations have become more vocal and open about their intimate thoughts and feelings (Yan, 2003), suggesting an expansion of norms around self-expression and emotion display compared to their elders. Compounded by a surging growth of cyberculture and Western influence, China is “unleashing emotions” (Lee, 2011). The transition to a market economy, for example, has fostered a much more competitive socioeconomic environment, one which requires people to be more assertive and self-expressive (Luo et al., 2013). Meanwhile, the norm of emotional concealment is gradually receding.

If indeed emotional concealment is losing its cultural importance, and if self-expression has a newfound centrality, then we might expect a changing pattern of depressive symptom reporting in China. In other words, compared to Kleinman (1982), where somatic symptoms predominated in patients’ presentation of distress, would recently recruited patients be more likely to endorse psychological symptoms? Although this direct comparison has not yet been reported in the literature, we can nonetheless infer changes in symptom presentation from recent studies. For example, comparing Chinese and Euro-Canadian depressed patients, Dere et al. (2013a) found that respondents in both groups were willing to spontaneously report depressed mood as a presenting problem. Ryder et al. (2008), meanwhile, found that the tendency of Chinese patients, relative to Euro-Canadian patients, to emphasize somatic symptoms of depression on a structured clinical interview was mediated by externally-oriented thinking (EOT)—operationalized as a general lack of interest in reflecting on and communicating about emotional experience. EOT has since been shown to correlate with endorsement of traditional Chinese values (Dere et al., 2012, 2013b).

With shifts in cultural values that reflect increasing individualism, we might expect changes toward a more self-focused thinking style, such as rumination, and increasing openness to the use of psychological language when reporting depressive symptoms. Moreover, increasing attention to internal psychological states might shape the very experience of depression, so that increasing number of people in China suffer from psychological symptoms when depressed (Ryder and Chentsova-Dutton, 2012). Culture in this view not only influences self-presentation and discourses surrounding depression, but gets under the skin to mold experience (Ryder and Chentsova-Dutton, 2015).

How then do we best understand the increasing prevalence rates of depression in China over the past few decades? We might further investigate the problematic aspects of a shift toward individualism, or toward a society with massive amounts of internal migration and socioeconomic dislocation—but we might also consider the rapidity of the change itself as problematic, and not necessarily (or not merely) the direction of the change. We might prefer an explanation that focuses on how depression, or psychosocial distress more generally, is presented. Changes in emotion norms may have prompted changes in which experiences are most salient, leading to shifts in symptom experience and expression. Changes in societal attitudes toward the mentally ill may have prompted changes in the stigmatization of depression, leading to shifts in which symptoms people are willing to discuss (Ryder and Chentsova-Dutton, 2012). Finally, rapid sociocultural change may have brought shifts in the training of mental health professionals, alterations in diagnostic practice, or improvements in research methods, all under the ever-increasing influence of mainstream Western psychiatry. At present, we do not have an empirical database that would allow us to disentangle these possibilities. What we do know is that a serious attempt to tackle this challenge will require a more nuanced understanding of rapid sociocultural change combined with sophisticated research methods designed to address change in a multilevel way. We conclude with some thoughts on how this might be done.

## Concluding Thoughts

Sociocultural transformation in China has been accompanied by increased individualism and depression. Can we then conclude that profound changes in the social realm have *caused* elevated levels of individualism and depression? In keeping with the idea of mutual constitution, we expect that changes at the culture-level exert effects at the mind-level, and vice versa (Ryder et al., 2011). Our review shows that China’s modernization is indeed accompanied by parallel shifts in individualistic values, parenting styles, self-expression norms, and the experience and expression of depression. While we acknowledge that our model points us to a complex picture in which causality is bidirectional, we believe in this case that a compelling causal narrative can be told about how a deliberate change of course in terms of economic structure and social organization has had psychological consequences. The data to definitively establish this narrative, however, have not yet been produced. In order to strengthen the thesis that rising individualism and depression in China are *consequences* of rapid modernization, the following two questions must be confronted. First, to what extent can these psychological consequences be attributed to sociocultural change rather than generational differences? Second, how unique are these consequences to China?

The idea that young people tend to hold cultural values and beliefs different from their parents or grandparents is not new. Indeed, one could argue that the observed value differences in the studies reviewed here are due to age differences and not sociocultural changes. Social psychologists have addressed similar problems. For example, to study changes in individualism, self-esteem, and narcissistic personality traits in American youth over the last several decades, Twenge (2008) proposed a cross-temporal meta-analytic method that examines psychological constructs of interest in similar-age samples collected at different points in time. Thus, instead of cross-sectional studies that offer a glimpse of individualistic values in different age groups, the cross-temporal method holds age and study design constant across time points and allows researchers to attribute observed value changes to sociocultural changes over time. Using this method, Twenge and Foster (2010) and Twenge (2015) found increasing self-reports of individualistic values, self-esteem, narcissism, and depression in same-age cohorts in the US between 1980 and the 2000s.

To our knowledge, with the exception of one life satisfaction study using WVS data across time (Steele and Lynch, 2013), the cross-temporal method has not been applied to investigate rising individualism in China. Therefore, we encourage researchers interested in the psychological consequences of rapid sociocultural change to either explore existing large survey data collected across time points or to start collecting crucial information about cultural values and mental health status in a systematic and consistent manner. Only then will we will be able to draw conclusions about rising individualism and depression with greater clarity and confidence.

Once we establish an association between sociocultural change and rising individualism and depression, we are confronted with a second question: to what extent are these psychological consequences unique to China? Recall that studies from the US and Japan, for example, have also shown links between sociocultural change and rising individualism (Twenge and Foster, 2010; Hamamura, 2012). To what extent might the *rapidity* of China’s modernization be central to shaping individualistic values and depression? To demonstrate the effects of *rapid* sociocultural change, researchers might consider comparing two or more cultural groups on variables of interest over time. A recent example would be Hamamura’s (2012) cross-temporal and cross-cultural analyses of individualism-collectivism in Japan and the US. This method could be applied to studies of rapid modernization. For example, one could compare changes in individualism and depression in China with other developing countries at varying rates of development across time points. The findings would allow us to examine differing trajectories of individualism and depression over time. More importantly, these results could help to generate hypothesis about the relation between rising individualistic values and depression. Several scholars have pointed out the link between modern living and increasing depression (e.g., Seligman, 1988; Hidaka, 2012). Twenge (2015), meanwhile, reported greater depressive symptoms and psychological distress in same-aged cohorts from the 2000s compared to those from the 1980s. Future studies should seek to unpack the mechanisms underlying the links between rapid modernization and depression in China by examining, for example, changes in the meaning of emotional expressiveness, externally vs. internally oriented thinking styles, and interpersonal relationships.

Finally, we encourage researchers to look beyond economic development as the sole indicator of cultural change. We anticipate that future studies will offer greater depth and breadth in understanding cultural and value change by including other culturally- and historically-relevant markers, such as family size (or one-child status), social and residential mobility, technology and the use of social media, international tourism, and degree of exposure to Western cultural contexts, or to ‘global culture’. For example, recent research on residential mobility has shown that people who have moved more times tend to emphasize the personal, individual self over the social, collective self (Oishi, 2010). Residential mobility may be one link between widespread sociocultural transformation in China and increased individualism, especially given the large rural-to-urban migration over the past few decades. Intriguingly, increased residential mobility is also associated with decreased well-being (Oishi et al., 2012), a link which may help future researchers to jointly consider rising individualism and falling well-being in China.

China’s ‘open door’ policy was much more than a shift toward a market-based economy. It was a shift toward integrating China and the Chinese people into the global flow of ideas. In his sociological review, Xie (2011) concludes that the scale and scope of the ‘Chinese transformation’ present many challenges, and opportunities, to the social scientist. We certainly believe this claim to be true for psychologists interested in the mutual constitution of culture, mind, and brain. Cultural psychologists have long argued that context is essential to understanding psychological processes, and have amassed a considerable amount of data documenting the degree to which culture shapes not only social behavior, but cognitive, emotional, and even neural processes (Markus and Hamedani, 2007; Kitayama and Uskul, 2011). Indeed, much of this work has been conducted with Chinese samples.

Studies conducted in rapidly changing societies highlight the extent to which psychological processes are shaped by the cultural-historical moment (Ryder et al., 2012). As with cultural psychology research more generally, documented shifts in psychological processes over relatively short periods of time challenge easy assumptions of psychological universality. At the same time, they provide an exciting opportunity for psychologists to study how culture shapes, and is shaped by, mind and brain. From that point of view, it is no wonder that Chairman Mao, and a contemporary Chinese factory worker, would hold radically different psychological perspectives on core values: after a mere 70 years, they inhabited radically different cultural worlds.

## Author Contributions

Both authors conceptualized the manuscript, JS wrote the first complete draft, AR contributed additional writing, both authors edited the manuscript and approved the final version.

## Conflict of Interest Statement

The authors declare that the research was conducted in the absence of any commercial or financial relationships that could be construed as a potential conflict of interest.

## Footnotes

1 The Chinese government announced the end of the One-Child Policy–and its replacement by a Two-Child Policy–as we were editing this manuscript, highlighting once more the rapidity of social change in China.

2 www.worldbank.org

3 Our use of ‘Western’ refers to people with cultural origins in European cultural contexts, with a particular emphasis on people living in the United States and Canada.

4 www.worldvaluessurvey.org
